# CT texture analysis in predicting treatment response and survival in patients with hepatocellular carcinoma treated with transarterial chemoembolization using random forest models

**DOI:** 10.1186/s12885-023-10620-z

**Published:** 2023-03-03

**Authors:** He An, Inderjeet Bhatia, Fei Cao, Zilin Huang, Chuanmiao Xie

**Affiliations:** 1 Diagnostic Imaging Division, Department of Medical Imaging and Interventional Radiology, Sun Yat-sen University Cancer Center, Guangzhou, China; 2 Department of Cardiothoracic Surgery, Queen Mary Hospital, Hong Kong, China; 3 Minimally Invasive Interventional Division, Department of Medical Imaging and Interventional Radiology, Sun Yat-sen University Cancer Center, Guangzhou, China; 4 Diagnostic Imaging Division, Department of Medical Imaging and Interventional Radiology, Sun Yat-sen University Cancer Center, 651 Dongfeng East Road, Guangzhou, 510060 China

**Keywords:** Computed tomography, Hepatocellular carcinoma, Random forest, Survival analysis, Texture analysis, Transarterial chemoembolization, Treatment response

## Abstract

**Background:**

Using texture features derived from contrast-enhanced computed tomography (CT) combined with general imaging features as well as clinical information to predict treatment response and survival in patients with hepatocellular carcinoma (HCC) who received transarterial chemoembolization (TACE) treatment.

**Methods:**

From January 2014 to November 2022, 289 patients with HCC who underwent TACE were retrospectively reviewed. Their clinical information was documented. Their treatment-naïve contrast-enhanced CTs were retrieved and reviewed by two independent radiologists. Four general imaging features were evaluated. Texture features were extracted based on the regions of interest (ROIs) drawn on the slice with the largest axial diameter of all lesions using Pyradiomics v3.0.1. After excluding features with low reproducibility and low predictive value, the remaining features were selected for further analyses. The data were randomly divided in a ratio of 8:2 for model training and testing. Random forest classifiers were built to predict patient response to TACE treatment. Random survival forest models were constructed to predict overall survival (OS) and progress-free survival (PFS).

**Results:**

We retrospectively evaluated 289 patients (55.4 ± 12.4 years old) with HCC treated with TACE. Twenty features, including 2 clinical features (ALT and AFP levels), 1 general imaging feature (presence or absence of portal vein thrombus) and 17 texture features, were included in model construction. The random forest classifier achieved an area under the curve (AUC) of 0.947 with an accuracy of 89.5% for predicting treatment response. The random survival forest showed good predictive performance with out-of-bag error rate of 0.347 (0.374) and a continuous ranked probability score (CRPS) of 0.170 (0.067) for the prediction of OS (PFS).

**Conclusions:**

Random forest algorithm based on texture features combined with general imaging features and clinical information is a robust method for predicting prognosis in patients with HCC treated with TACE, which may help avoid additional examinations and assist in treatment planning.

**Supplementary Information:**

The online version contains supplementary material available at 10.1186/s12885-023-10620-z.

## Background

Hepatocellular carcinoma (HCC) is a malignant disease with high mortality. Many risk factors have been well established that impact the outcomes of HCC, including age, gender, staging, ascites, tumour thrombus and liver function [1]. Curative surgery will increase the long-term survival rate. However, not all HCCs can be treated with surgical resection due to the high disease burden, insufficient residual liver volume, severe cirrhosis, disseminated metastatic lesions within the liver, presence of portal vein tumour thrombus and other cancer-related symptoms [2].

The Barcelona Clinic Liver Cancer (BCLC) staging system supported transarterial chemoembolization (TACE) as the first treatment choice in patients with unresectable HCC, such as those with large or multinodular HCC. The same recommendation is also made in the Chinese University Prognostic Index (CUPI) [3] and the Hong Kong Liver Cancer (HKLC) staging system [4]. The long-term survival was prolonged in patients with unresectable HCC when treated with TACE compared to the best supportive care [5]. However, in the clinical setting, the therapeutic outcome of TACE is not always satisfying when it comes to individual cases because the biological behaviour of tumour cells is highly heterogeneous.

Modified Response Evaluation Criteria In Solid Tumors (mRECIST) is a criterion relying on the change of tumour burden before and after treatment in HCC [6]. Several studies have demonstrated that the objective response assessed by mRECIST is independently prognostic for survival and can be considered a valid endpoint in HCC clinical trials [7–9].

Currently, the assessment mainly depends on imaging methods, such as computed tomography (CT) and magnetic resonance imaging (MRI). Though many image characteristics have been suggested as having prognostic value, substantial subtle features were omitted during traditional imaging assessment, which is highly dependent on individual experience and limited by human eye resolution. CT texture analysis is a post-processing algorithm that further defines tumour characteristics beyond the perception of human eyes. By conducting texture analysis, large amounts of texture features are extracted from the pre-treatment images, which can reflect tumour heterogeneity, showing both morphological and cellular diversity [10]. It has been widely applied in many cancer types to predict patient outcome [11–14].

Thus, the primary aim of this study was to create a robust model incorporating texture features derived from contrast-enhanced CT combined with general imaging features as well as clinical information to predict treatment response. Secondary analyses aimed to determine the features that predicted the overall survival (OS) and progress-free survival (PFS) in patients with HCC who received TACE.

## Methods

### Patients

This study was approved by the Sun Yat-sen University Cancer Centre Institutional Review Board (No. B2021-214–01) with a waiver of written informed consent. All methods were carried out in accordance with relevant guidelines and regulations. From January 2014 to November 2022, data on patients with histological diagnoses of HCC were retrieved from our centre's database. Inclusion criteria were patients (1) with contrast-enhanced CT of the abdomen performed before the initiation of treatment; (2) who received TACE treatment, and (3) who had 1^st^ follow-up CT within 4–6 weeks after TACE. Exclusion criteria included patients (1) with a single lesion with a maximal diameter of less than 1 cm or not detectable on baseline CT; (2) having disseminated disease within the liver precluding the placement of regions of interest (ROIs); (3) received other treatments before or after TACE, including surgery, radiofrequency treatment or liver transplantation; (4) with no corresponding laboratory test results, and (5) with other malignancies. Patient demographics were recorded, including age, gender, BCLC stage, Child − Pugh class, Eastern Cooperative Oncology Group (ECOG) performance status and complications (diabetes or hypertension). Laboratory test results, including platelet (PLT) count, alanine transaminase (ALT), aspartate aminotransferase (AST), total bilirubin (TBIL), international normalized ratio (INR), alkaline phosphatase (ALP), albumin (ALB), C-reactive protein (CRP), alpha-fetoprotein (AFP), hepatitis B virus (HBV) and hepatitis C virus (HCV) were collected.

### CT acquisition

CT examinations were performed using 2 scanners with intravenous contrast media. The volume of the contrast media was determined by multiplying the body weight (in kilograms) by 2 to a maximum of 100 mL. The concentration of the iodinated contrast media was 350 mg/mL with an injection rate of 2 mL/s. The scanning parameters of the 2 scanners were as follows: (1) The 128-channel CT scanner (Discovery CT750, GE Healthcare, US): field of view, 25 cm; matrix, 512 × 512; tube voltage, 120 kVp; tube current, 200–400 mA; reconstructed thickness, 5 mm; (2) The 128-channel CT scanners (Somatom Definition or Definition AS + , Siemens Healthcare, US): field of view, 35 cm; matrix, 512 × 512; tube voltage, 80–120 kVp; tube current, 248–578 mA; reconstructed thickness, 5 mm. Finally, the arterial phase images of the CT examination were anonymized and assigned a research code for the assessment of general imaging features and the extraction of texture features.

### General imaging features assessment

All data were reviewed by 2 board-certified radiologists on a dedicated software (ITK-SNAP, v 3.8.0). The senior radiologist (R1) had more than 10 years of cross-sectional imaging experience, while the junior radiologist (R2) had 5 years of cross-sectional imaging experience. This was designed to test for inter-observer agreement. Only the data from the senior radiologist was used for subsequent feature extraction and model construction.

To begin with, they identified all lesions for each patient in consensus and marked the slice of the largest axial diameter of each lesion. Then, they evaluated general imaging features and drew ROIs separately. Four general imaging features were assessed, including (1) largest tumour diameter, (2) number of lesions, (3) presence or absence of portal vein thrombus, and (4) presence or absence of ascites. Univariate and multivariate logistic regression was conducted to select clinical features that had independent prognostic value.

### ROI delineation and Texture feature extraction

ROIs were drawn by strictly delineating around the margin of the mass with careful inclusion of both solid and cystic components but exclusion of adjacent normal structures (Suppl 1). If there were multiple lesions, all would be given an ROI delineation.

Texture feature extraction was performed on an open-source Python-based radiomics software (PyRadiomics, v 2.2.0). First, all images are normalized and scaled before textual computation. Then, 5 filters were applied, including Laplacian of Gaussian, wavelet, square, square root, logarithm, and exponential filters [15]. Wavelet transformation was applied with a single-level directional discrete wavelet transform of high-pass and low-pass filters [16]. Eight wavelet-decomposition images were created, including HHH, HHL, HLH, HLL, LHH, LHL, LLH, and LLL (H: high-pass filter; L: low-pass filter). Finally, 1618 texture features were extracted, including (1) first-order statistics, (2) shape-based features, (3) gray-level co-occurrence matrix (GLCM), (4) gray-level-dependent matrix (GLDM), (5) neighboring gray tone difference matrix (NGTDM), (6) gray-level size zone matrix (GLSZM), and (7) gray-level run length matrix (GLRLM). More details about these features were tabulated in Table 1 [17].

**Table 1:** Description of texture feature groups

Texture feature group	Description
(1) First-order statistics	Distribution of grey-level intensities
(2) Shape-based features	Description of two- and three- dimensional shape and size
(3) Gray-level co-occurrence matrix (GLCM)	The spatial relationship of pixel intensities
(4) Gray-level-dependent matrix (GLDM)	Gray level dependencies independent from angles
(5) Neighboring gray tone difference matrix (NGTDM)	Difference between gray-level and the average within certain distances
(6) Gray-level size zone matrix (GLSZM)	Description of the size of homogeneous zones for each grey-level in 3 dimensions
(7) Gray-level run length matrix (GLRLM)	The number of pairs of gray level value and its length of runs

### Feature reduction and selection

**Fig. 1:**
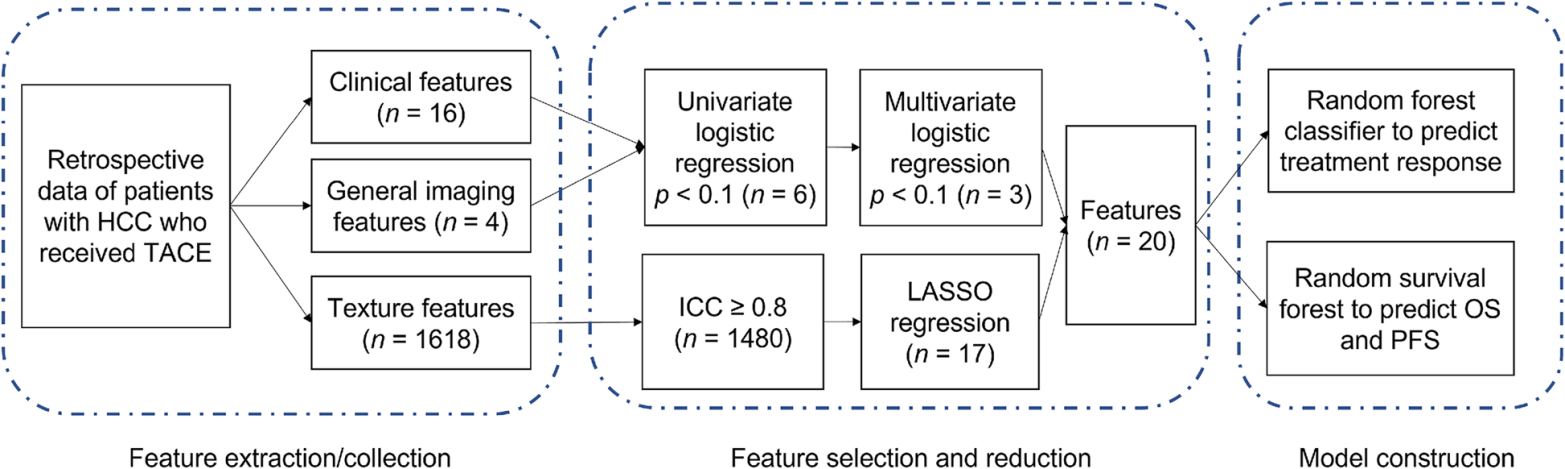
A flowchart depicting the analytical procedures. HCC = hepatocellular carcinoma; ICC = intraclass correlation coefficient; LASSO = least absolute shrinkage and selection operator; OS = overall survival; PFS = progress-free survival; TACE = transarterial chemoembolization

First, all texture features were tested by the intraclass correlation coefficient (ICC). Features with low inter-rater reproducibility (ICC < 0.8) were excluded. Next, the least absolute shrinkage and selection operator (LASSO) algorithm was employed for further feature reduction. The tuning parameter (λ) was selected using 10-fold cross-validation and minimum criteria. A plot of the partial likelihood deviance was made against log (λ). The minimum (lambda.min) and 1-SE criteria (lambda.1se) were used to draw the dotted vertical lines at the optimal values. The whole analytical procedure was shown in Fig. 1.

### TACE procedures

Patients were given TACE using cytotoxic drugs as determined by a local multi-disciplinary team in accordance with the recommendations of the European/American Association for Liver Disease guidelines [18, 19].

Conventional TACE was performed through femoral access under moderate sedation using the Seldinger technique [20]. To cause embolization of the tumour microcirculation, cytotoxic drugs or chemotherapeutic agents suspended in lipiodol were administrated into the tumour-feeding artery with a dose ranging from 5 to 30 mL depending on the location, the size, and the number of lesions. If necessary, gelatin sponge particles (150–350 μm) were injected to block the blood until the flow was static.

### Assessment of treatment response and follow-up

All patients had 1^st^ follow-up CT within 4–6 weeks after TACE. Their treatment response was evaluated by mRECIST. Patients were dichotomized into the progress-free cohort, including those who achieved complete response (CR), partial response (PR), stable disease (SD), and the progress cohort, including those who exhibited progressive disease (PD) during follow-up. CR was defined as no intratumorally arterial enhancement in all target lesions. PR was defined as at least a 30% decrease in the sum of diameters of viable (enhancement of arterial phase) target lesions taking as reference the baseline sum of the diameters of target lesions. SD was defined as neither PR nor PD. PD was defined as an over 20% increase in the sum of the diameters of viable arterial-enhancing target lesions or new nodule formation.

All patients were followed up by telephone or clinical visits once every 2 months during the first year and once every 3 months after that until death or the last follow-up day (30th November 2022). OS was defined as the time from baseline CT to death or censoring date. PFS was defined as the time from TACE to disease progression based on mRECIST, death, or censoring date.

### Statistics

Data were described as mean and standard deviation or median and range tested by the Shapiro–Wilk test. Fisher’s exact test and Welch’s T-test were used to verify differences among features. The Dice coefficient was calculated between the ROIs drawn by the two radiologists. Univariate and multivariate logistic regression was used to select clinical and general imaging features. ICC and LASSO regression was used to select texture features.

The data were randomly divided in a ratio of 8:2 for model training and testing. To test the added value of texture features to the predictive model, two random forest classifiers (Model 1 including selected clinical information, general imaging features and texture features; Model 2 including selected clinical information and general imaging features) were created to differentiate the progress-free cohort from the progress cohort. Random survival forest models (Model 1 including selected clinical information, general imaging features and texture features; Model 2 including clinical information and general imaging features) were used to evaluate OS and PFS in patients with HCC treated with TACE. Statistical analysis was conducted using R version 3.5.1 (R Foundation for Statistical Computing, Vienna, Austria). The "randomForest” and “randomForestSRC” packages were implemented. A *p* < 0.05 was considered statistically significant.

## Results

### Demographics

A total of 289 patients with HCC who received TACE treatment were retrospectively included in this study, with an average age of 55.4 ± 12.4 years. Most of them (*N* = 261, 90.3%) were male. The median time interval from baseline CT examination to TACE treatment was 4.5 days (range: 1–14 days) and from TACE treatment to 1^st^ follow-up CT examination was 35 days (range: 28–42 days). Patients were randomly allocated into training and testing sets in the ratio of 8:2 for analytical purposes. Detailed patient characteristics were reported in Table 2.

**Table 2:** Patient demographics

		Whole cohort	Progress-free cohort	Progress cohort	*p*
*N*		289	224	65	
Age (years)		55.4 ± 12.4	54.6 ± 12.5	58.3 ± 11.6	**0.038**
Gender	Male	261	204 (78.2%)	57 (21.8%)	0.417
	Female	28	20 (71.4%)	8 (28.6%)	
ECOG performance status	0	286	223 (78.0%)	63 (22.0%)	0.128
	1	3	1 (33.3%)	2 (66.7%)	
Complications	0	244	190 (77.9%)	54 (22.1%)	0.733
	1	45	34 (75.6%)	11 (24.4%)	
Hepatitis	0	48	40 (83.3%)	8 (16.7%)	0.290
	1	241	184 (76.3%)	57 (23.7%)	
ALB (g/L)		39.1 ± 4.7	39.1 ± 4.6	39.1 ± 5.0	0.998
ALT (u/L)*		46.0 (9.7–1053.7)	39.2 (15.8–140.9)	49.8 (9.7–1053.7)	**0.025**
AST (u/L)*		64.7 (12.6–2680.0)	51.8 (17.7–323.7)	68.2 (12.6–2680.0)	0.085
TBIL (μmol/L)		17.2 ± 9.2	16.0 ± 8.4	17.6 ± 10.7	0.273
PT (s)		12.2 ± 1.1	12.1 ± 1.1	12.3 ± 1.2	0.337
INR		1.1 ± 0.1	1.1 ± 0.1	1.1 ± 0.1	0.216
PLT (× 10^9^/L)		222.9 ± 107.8	206.4 ± 110.9	227.7 ± 102.2	0.147
Child–Pugh class	A	272	209 (76.8%)	63 (23.2%)	0.220
	B	17	15 (88.2%)	2 (11.8%)	
CRP (mg/L)		23.8 ± 34.4	22.2 ± 31.1	27.6 ± 41.2	0.330
BCLC stage	A	135	115 (85.2%)	20 (14.8%)	**0.013**
	B	92	66 (71.7%)	26 (28.3%)	
	C	62	43 (69.4%)	19 (30.6%)	
AFP (μg/L)*		700.3 (1.1–865,569.0)	83.4 (1.1–121,000.0)	972.8 (1.62–865,569.0)	**0.012**

ECOG 0 = fully active; 1 = restricted in physically strenuous activity

Complications 0 = without any complications; 1 = with complications (diabetes or hypertension)

Hepatitis 0 = without hepatitis; 1 = with hepatitis B or with hepatitis C

Child–Pugh score A = liver is functioning well; B = mild or moderate cirrhosis

BCLC staging A = early stage, a single tumour of any size, or up to 3 tumours all less than 3 cm; B = multiple tumours in the liver; C = metastasis to the blood vessels, lymph nodes or other body organs

^*^ Non-normal distribution summarised as median and range

### Clinical and general imaging features selection

All clinical features (age, gender, ECOG performance status, Complications, Hepatitis, ALB, ALT, AST, TBIL, PT, INR, PLT, Child–Pugh class, CRP, BCLC stage, AFP) and general imaging features (diameter of the largest lesion, number of lesions, presence or absence of portal vein thrombosis, and presence or absence of ascites) were included in univariate logistic analysis. Detailed results of the assessment of general imaging features were tabulated in Suppl 2. Those significant features were included in multivariate logistic analysis (Table 3). Finally, 2 clinical features (ALT, AFP levels) and 1 general imaging feature (presence or absence of portal vein thrombus) were selected for further model construction.

**Table 3:** Univariate and multivariate logistic regression of clinical features and general imaging features in the classification of short-term treatment response

Feature	Univariate		Multivariate	
	Odds ratio (95% confidence interval)	*p*	Odds ratio (95% confidence interval)	*p*
**Clinical features**				
Age	0.976 (0.954–0.999)	0.038	0.991 (0.996–1.017)	0.482
ALT	1.010 (1.002–1.019)	0.025	1.011 (1.003–1.020)	**0.008**
BCLC stage	1.622 (1.146–2.295)	0.013	0.935 (0.576–1.519)	0.786
AFP	1.047 (1.003–1.091)	0.012	1.066 (1.010–1.121)	**0.030**
**General imaging features**				
Presence of portal vein thrombus	3.615 (1.795–7.283)	< 0.001	3.305 (1.307–8.355)	**0.012**
Presence of ascites	4.500 (1.043–19.423)	0.028	4.085 (0.912–18.306)	0.066

### Texture features reduction and selection

The mean Dice similarity coefficient of the ROIs between two radiologists was 0.90 ± 0.08. A total of 1618 texture features were extracted for each patient. First, the features with low inter-rater reproducibility (ICC < 0.8) were excluded, thus reducing the number of texture features to 1480. Next, the LASSO algorithm was conducted to select features that had prognostic value. The minimum (lambda.min) and 1-SE criteria (lambda.1se) were 0.019 and 0.070, respectively (Suppl 3). Finally, 17 texture features were included in further model construction.

### Treatment response

During the follow-up, a small proportion of patients (22.5%) achieved progressive outcomes (PD *N* = 65), while the rest (77.5%) had progress-free disease (CR* N* = 1, PR *N* = 139 and SD *N* = 84). The patients were dichotomized into progress-free and progress cohorts for analytic purposes.

**Fig. 2:**
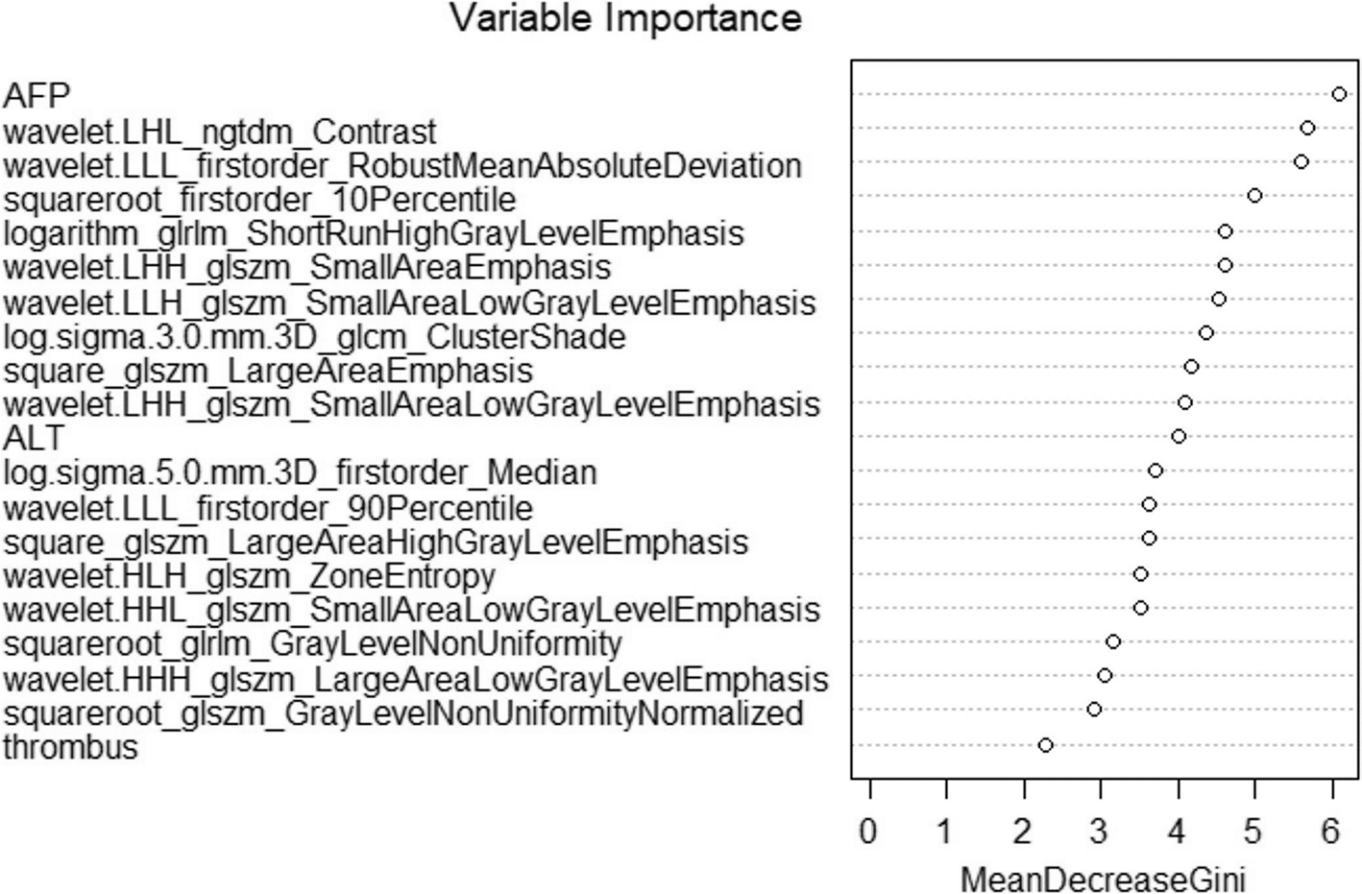
The Gini importance of random forest classifier based on selected features for the assessment of treatment response. AFP = alpha-fetoprotein; ALT = alanine transaminase

There was no significant difference between training and testing sets for all selected features (*p* > 0.05). Our random forest Model 1 based on the selected 2 clinical features (AFP and ALT levels), 1 general imaging feature (presence or absence of portal vein thrombus) and 17 texture features resulted in an AUC of 0.947 with a 95% confidence interval (CI) of 0.889–1.000 for predicting treatment response after TACE. The accuracy was 89.5% (95% CI: 78.5%-96.0%). The top 3 features for predicting mRECIST were AFP level, texture features wavelet.LHL_ngtdm_Contrast, and wavelet.LLL_firstorder_RobustMeanAbsoluteDeviation. Further model performance was tabulated in Table 4 and Suppl 4. The Gini importance was plotted in Fig. 2.

**Table 4:** Performance of random forest classifier of Model 1 incorporating selected clinical and general imaging features with texture features and Model 2 incorporating selected clinical and general imaging features without texture features (*p* < 0.001)

	Model 1		Model 2	
	Training set (95% confidence interval)	Testing set (95% confidence interval)	Training set (95% confidence interval)	Testing set (95% confidence interval)
Sensitivity (%)	100.0 (97.3–100.0)	68.8 (41.5–87.9)	14.8 (7.1–27.7)	12.5 (2.2–39.6)
Specificity (%)	100.0 (92.0–100.0)	97.6 (85.6–99.9)	100.0 (97.4–100.0)	97.6 (85.6–99.9)
Positive predictive value (%)	100.0 (97.3–100.0)	91.7 (59.8–99.6)	100.0 (59.8–100.0)	66.7 (12.5–98.2)
Negative predictive value (%)	100.0 (92.0–100.0)	88.9 (75.2–95.8)	79.5 (73.5–84.4)	74.1 (60.0–84.6)
Accuracy (%)	100.0 (98.4–100.0)	89.5 (78.5–96.0)	80.2 (74.5–85.1)	73.7 (60.3–84.5)
Area under the curve	1.000 (1.000–1.000)	0.947 (0.889–1.000)	0.971 (0.953–0.990)	0.867 (0.765–0.968)

Another random forest Model 2 based on the selected 2 clinical features (ALT and AFP levels) and 1 general imaging feature (presence or absence of portal vein thrombosis) resulted in an AUC of 0.867 with a 95% confidence interval (CI) of 0.765–0.968 for predicting treatment response after TACE. The accuracy was 73.7% (95% CI: 60.3%-84.5%), significantly inferior to Model 1 (*p* < 0.001).

### Survival analysis

The median follow-up period was 38 months. On the last follow-up day, 121 alive patients were documented as censored for the OS analysis. Sixty-five patients without progressive disease were documented as censored for PFS analysis. The median OS and PFS and were 281 (range: 47–2578) days and 54 (range: 5–1074) days, respectively.

**Fig. 3:**
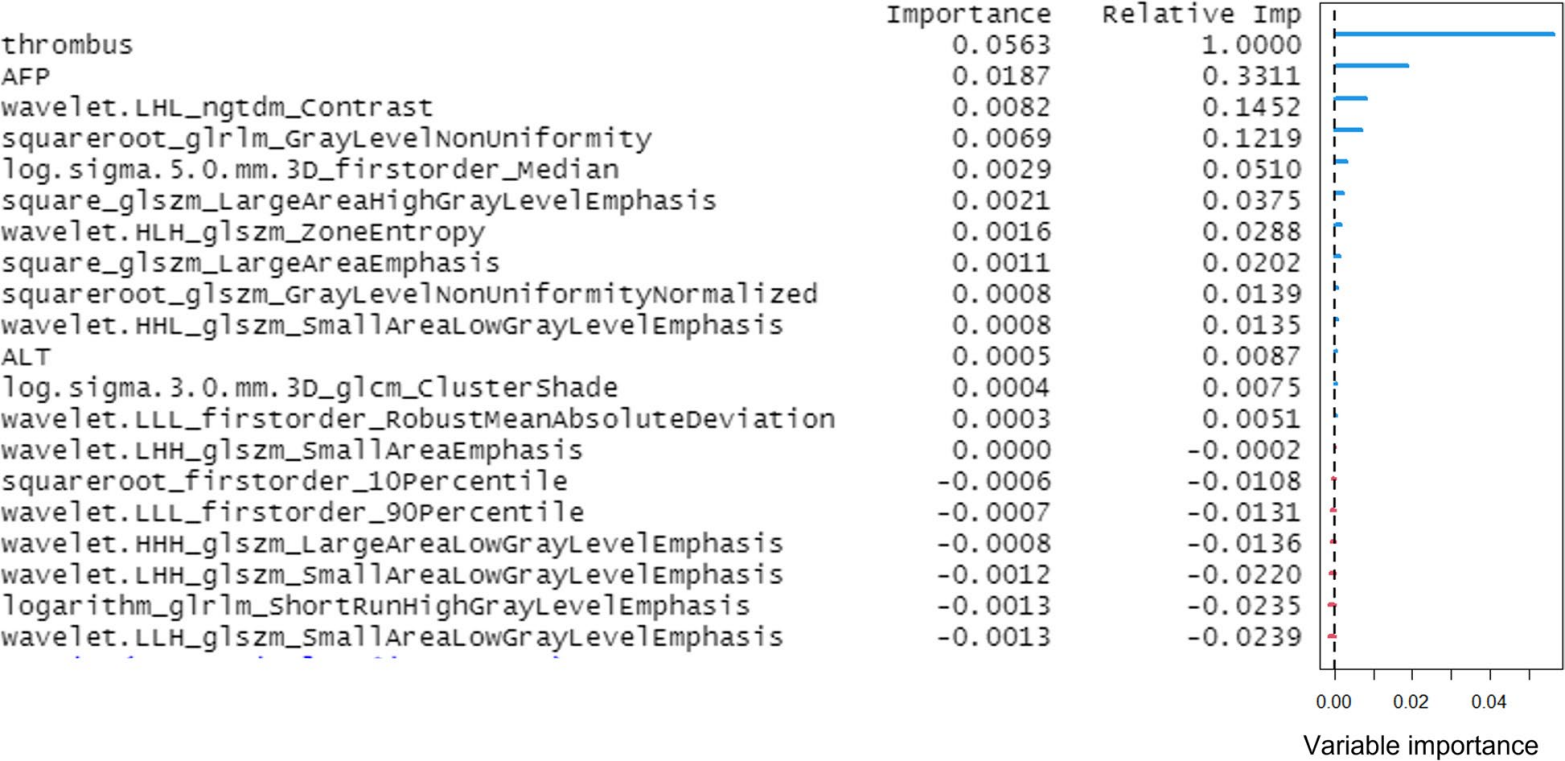
Importance list of 20 features in the random survival forest predicting OS. The abscissa depicts the variable importance. Relative importance is calculated by dividing each variable importance score by the largest importance score of the variables. AFP = alpha-fetoprotein; ALT = alanine transaminase; OS = overall survival

**Fig. 4:**
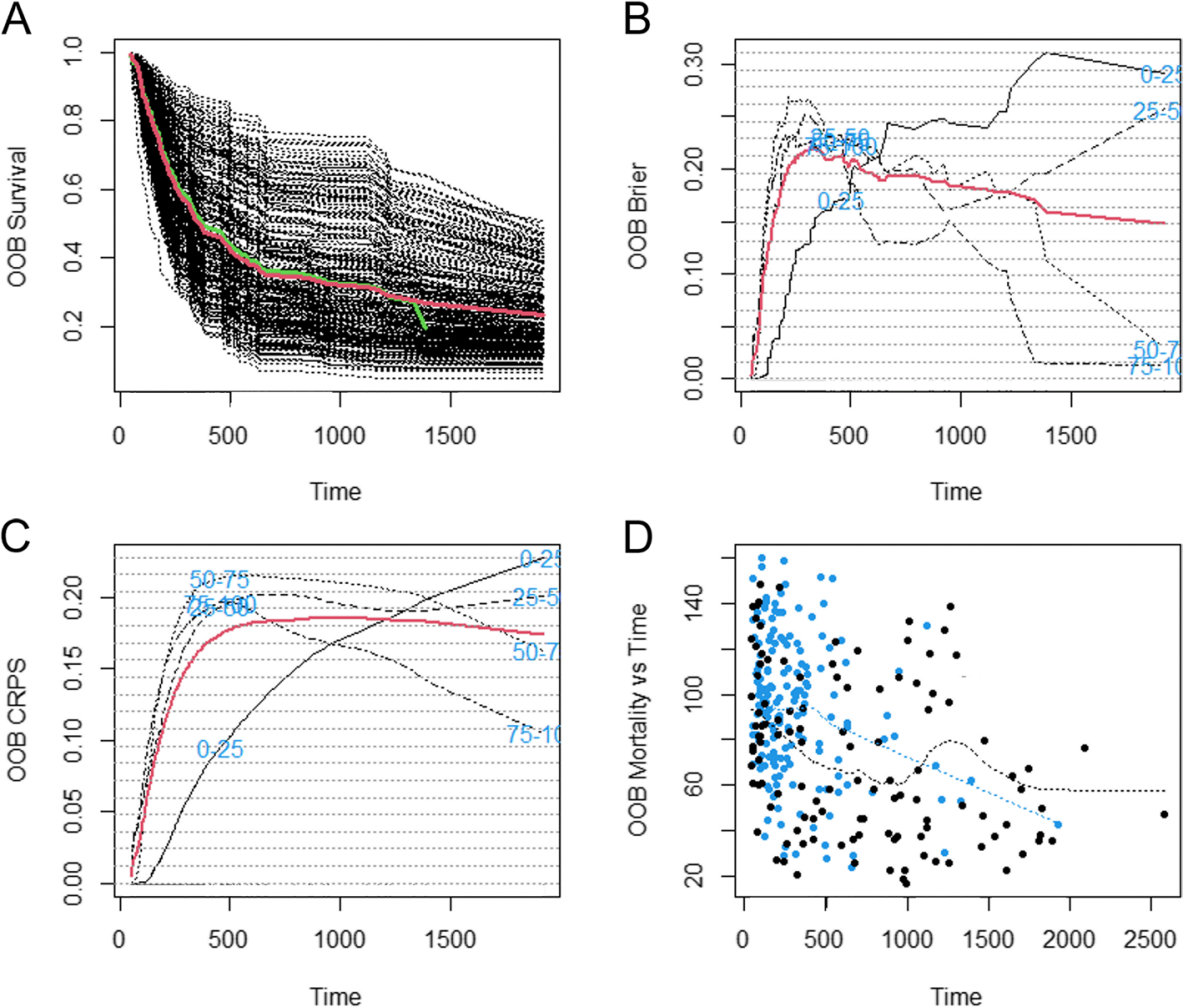
Performance of random survival forest predicting OS over time (days). **A** Forest estimated survival function. The black line indicates each individual; the thick red line indicates overall ensemble survival and the thick green line indicates the Nelson-Aalen estimator. **B** Brier score stratified by ensemble mortality. Red line is the overall (non-stratified) Brier score. This score ranges from 0–1 (0 = perfect, 1 = poor); **C** The CRPS was calculated as Brier score divided by time; **D** Plots of mortality of each individual versus observed time. Points in blue correspond to death events, black points are censored observations. CRPS = continuous ranked probability score; OS = overall survival

The selected 20 features were included in the random survival forest to predict OS in patients with HCC treated with TACE. In Model 1, the random forest algorithm, including 2 clinical features (AFP and ALT levels), 1 general imaging feature (presence or absence of portal vein thrombus) and 17 texture features achieved OOB Error Rate of 0.347 to predict OS. The continuous ranked probability score (CRPS) was 0.170. The presence or absence of portal vein thrombus, AFP level and texture feature wavelet.LHL_ngtdm_Contrast were the top 3 features of importance (Fig. 3). In addition, the plot of the time-dependent OOB Brier score and CRPS demonstrated that the random survival forest model for the prediction of OS performed particularly well in the first year, especially for the low-risk quartile sample set (0–25% line) and continued to perform well afterwards (Fig. 4). In Model 2, the random forest algorithm 2, including clinical features (AFP and ALT levels) and 1 general imaging feature (presence or absence of portal vein thrombus) achieved OOB Error Rate of 0.576. The CRPS was 0.282, which is inferior to Model 1.

**Fig. 5:**
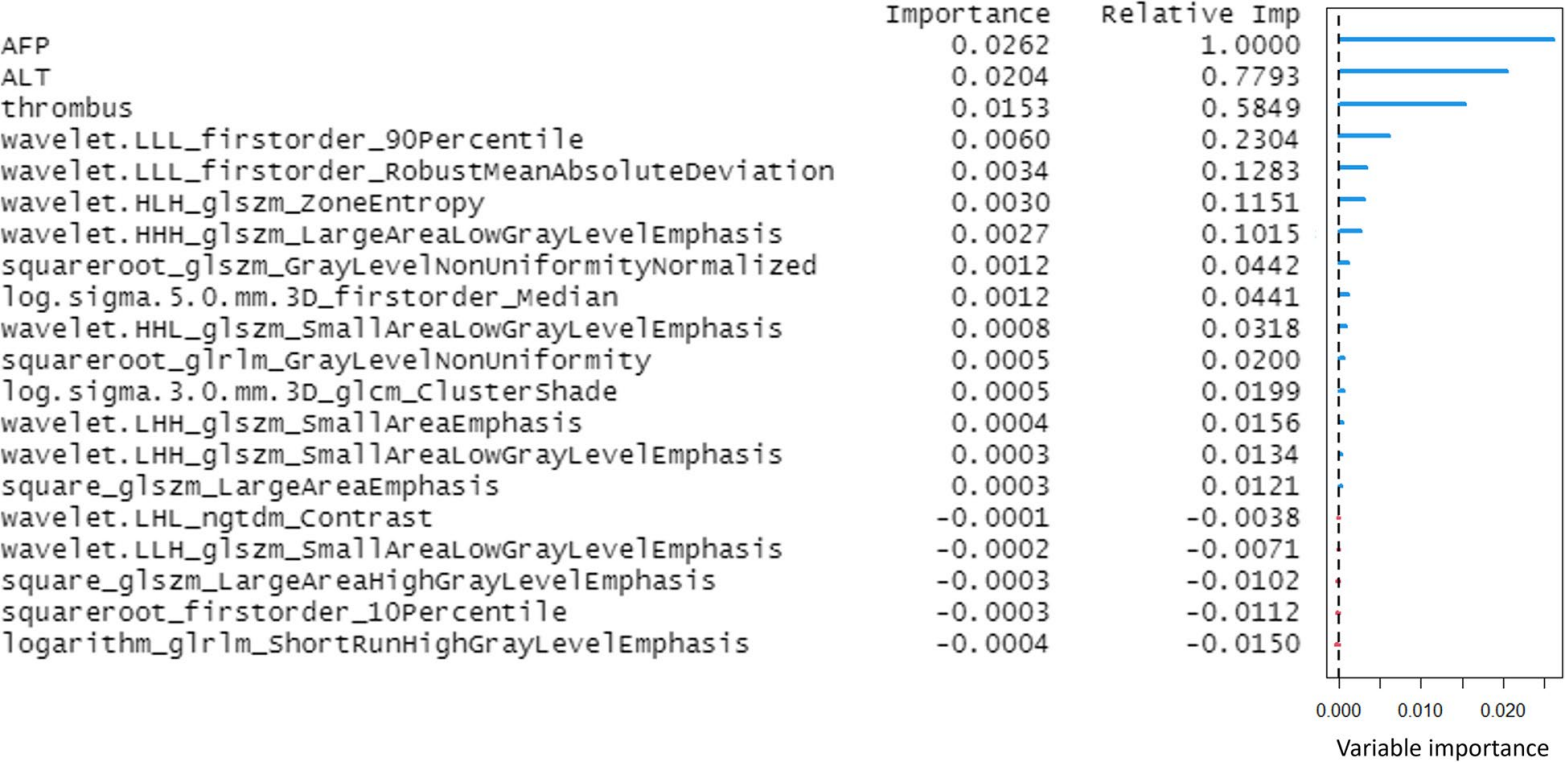
Importance list of 20 features in the random survival forest predicting PFS. The abscissa depicts the variable importance. Relative importance is calculated by dividing each variable importance score by the largest importance score of the variables. AFP = alpha-fetoprotein; ALT = alanine transaminase; PFS = progress-free survival

**Fig. 6:**
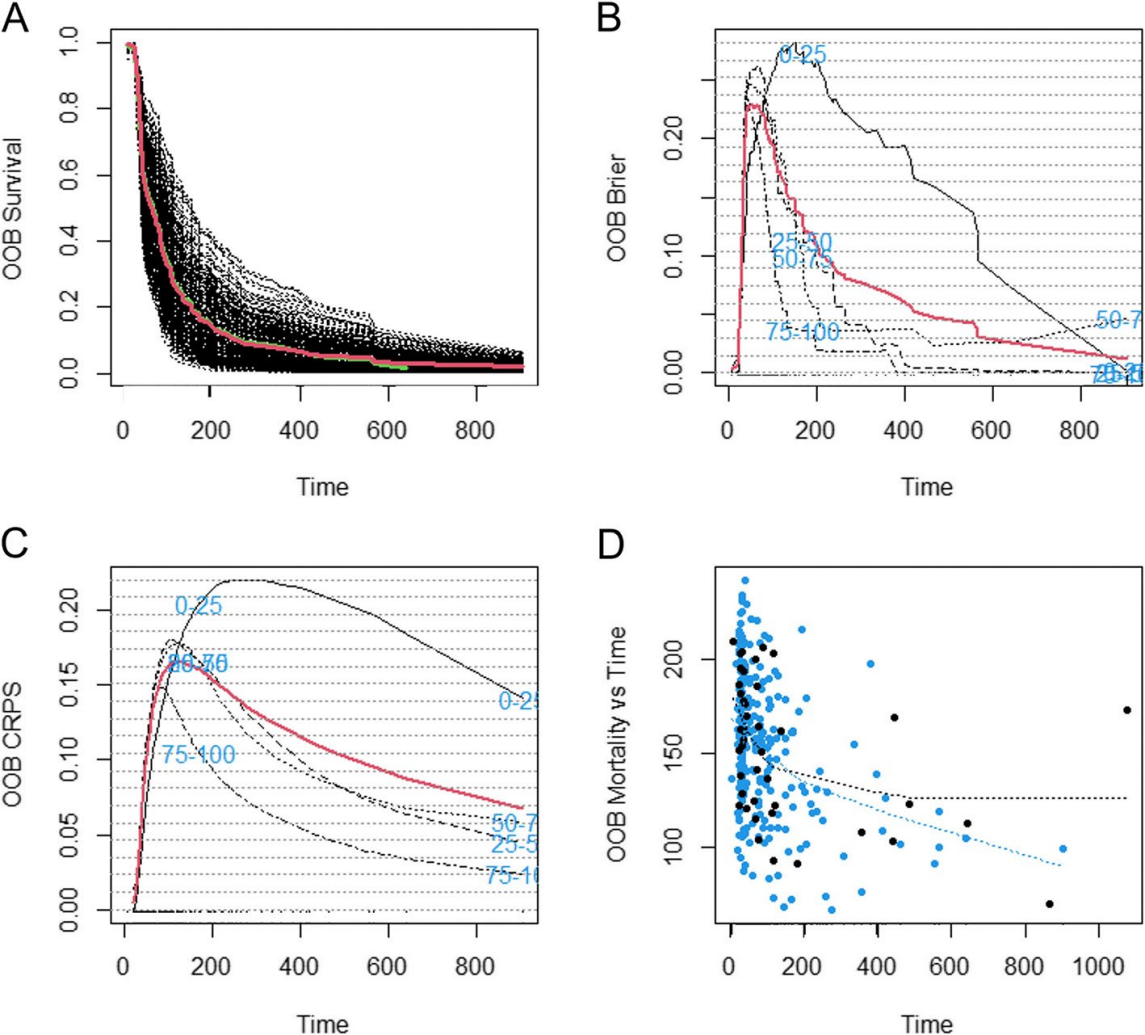
Performance of random survival forest predicting PFS over time (days). **A** Forest estimated survival function. The black line indicates each individual; the thick red line indicates progress-free ensemble survival and the thick green line indicates the Nelson-Aalen estimator. **B** Brier score stratified by ensemble mortality. Red line is the progress-free (non-stratified) Brier score. This score ranges from 0–1 (0 = perfect, 1 = poor); **C** The CRPS was calculated as Brier score divided by time; **D** Plots of mortality of each individual versus observed time. Points in blue correspond to disease progression, black points are censored observations. CRPS = continuous ranked probability score; PFS = progress-free survival

In Model 1, the random survival forest algorithm achieved OOB Error Rate of 0.374 to predict PFS. The CRPS was 0.067. AFP level, ALT level and presence or absence of portal vein thrombus were the top 3 features of importance (Fig. 5). In addition, the plot of the time-dependent OOB Brier score and CRPS demonstrated that the random survival forest model for the prediction of PFS performed the worst for the low-risk quartile sample set (0–25% line) and had better performance over long term compared to the first 200 days (Fig. 6). In Model 2, the random forest algorithm achieved OOB Error Rate of 0.529. The CRPS was 0.193, which is inferior to Model 1.

## Discussion

Our study constructed a random forest classifier that incorporated CT texture features, general imaging features and clinical information in predicting treatment response in HCC after TACE. This model achieved good performance, offering an objective and non-invasive method for evaluating TACE treatment, potentially avoiding extra imaging examinations or diagnostic work-up and facilitating personalized treatment.

It has been well established that high tumour burden, impaired liver function, incomplete necrosis, the occurrence of extrahepatic spread and vascular invasion should reduce the therapeutic effectiveness of TACE [21]. The AFP level was reported as one of the most significant prognostic factors to predict treatment outcome, and the changes in AFP after treatment highly correlated with radiologic response and survival [22, 23]. Similar conclusions have been drawn from our study, in which AFP level showed high importance for predicting mRECIST, OS and PFS. Elevated ALT level indicates the impairment of liver function and is a critical point when assessing TACE feasibility. Interestingly, ALT showed high importance in the prediction of PFS in our study. This phenomenon could be preserved liver function will help form sufficient tumour necrosis after TACE which prevented tumour invasion and progression. The presence of portal vein thrombus has been well established as a negative predictor for survival [24]. We observed that the presence of thrombus had high importance in OS and PFS random survival forest models, in contrast, for the prediction of mRECIST, the presence of portal vein thrombus was the least important variable. This could be due to the fact that following TACE, immediate treatment response was rapidly assessed, while the presence of portal vein thrombus may have a long-term adverse effect on prognosis.

A series of studies have been published using texture analysis or radiomic techniques to predict the response of TACE in HCC across different modalities, such as non-contrast CT [25], contrast CT [26], and MRI [27], with good performance (AUC ranging from 0.884–0.960). Tumour heterogeneity and size were identified as critical prognostic features in the Nested multiparametric decision tree [28]. The histogram-based features and shape features were reported to be sensitive in determining the nature of the tumour, which is related to tumoral heterogeneity [29, 30]. Our study achieved a similar or higher AUC compared to the above-mentioned studies and several texture features showed high importance in our classifier, especially for the 3-dimension grey level in a matrix classified by region volume, the GLSZM. These features indirectly express a higher degree of tissue homogeneity, which may be interpreted as a consequence of the TACE treatment response, thus reducing the contrast between neighbouring voxels in the non-tumoral component. The CT textural analysis markedly added to the information generated by the clinical parameters in the model for the prediction of immediate treatment response after TACE.

Our random survival forest Model 1 incorporating texture features showed lower CRPS and OOB Error Rate compared to Model 2 based on clinical information and general imaging features. Thus, CT texture features had added value in predicting survival in patients with HCC treated with TACE. Prior studies have proven the value of several features in predicting survival in HCC, including AFP level, ALT level, and presence or absence of portal vein thrombosis [31–34], similarly those features showed high importance in our random survival forest models. There are two main differences between our study and the prior studies. First, our study extracted several texture features to characterize tumour nature and demonstrated high importance. Second, prior studies constructed models based on radiomics or clinical scores that might have dependencies. Their combination without considering the potential correlation among these dependencies might lead to overfitting the data, in contrast, the random forest algorithms in our study can prevent overfitting by simply reducing tree depth.

There were limitations in our study. First, only arterial phase images were analysed in our study as previous studies have proven that the extracellular volume and blood flow in HCC during the arterial phase could give rise to unique radiological features [35–37]. Second, selection bias could have resulted from the fact that the patients were recruited from a single specialized oncology medical centre, and that by the time they sought treatment here, their disease may have already been advanced. Finally, the results from this study were based on texture features extracted using one software. They may not be applicable when using other platforms with different analysis algorithms or higher-order statistics. Standardization and data reproducibility are important before CT texture analysis can be widely applied in clinics.

## Conclusions

The current study showed that using random forest algorithms based on the combination of clinical information, general imaging features and texture features derived from pre-treatment contrast-enhanced CT could predict treatment response and survival in HCC treated with TACE. Our findings potentially help patients with HCC avoid additional examinations and assist in treatment planning.

## Supplementary Information

**Additional file 1:** **Suppl 1. **Contrast-enhanced CT in a 65-year-old male with HCC. The red mask is the demonstration of ROIs placement on the axial phase image. **Suppl 2.** Summary of general imaging features. **Suppl 3. **Feature selection using the LASSO algorithm. (A) Selection of the tuning parameter (λ) using 10-fold cross-validation and the minimum criteria. A plot of the partial likelihood deviance was made against log (λ). The minimum and 1-SE criteria were used to draw the dotted vertical lines at the optimal values. (B) Profiles of the LASSO coefficients for the texture features. The vertical line was drawn at a value selected from the log (λ) sequence using 10-fold cross-validation. Seventeen texture features were selected within this range. **Suppl 4.** ROC curves of Model 1 (A) incorporating selected clinical information, general imaging features and texture features and Model 2 (B) without texture features for the prediction of treatment response.

## Data Availability

The anonymized original data is available from the corresponding author, upon reasonable request.

## Abbreviations

ALT: Alanine transaminase
ALB: Albumin
ALP: Alkaline phosphatase
AFP: Alpha-fetoprotein
AUC: Area under the curve
AST: Aspartate aminotransferase
BCLC: Barcelona Clinic Liver Cancer
CUPI: Chinese University Prognostic Index
CR: Complete response
CT: Computed tomography
CI: Confidence interval
CRPS: Continuous ranked probability score
CRP: C-reactive protein
ECOG: Eastern Cooperative Oncology Group
GLCM: Gray-level co-occurrence matrix
GLRLM: Gray-level run length matrix
GLSZM: Gray-level size zone matrix
GLDM: Gray-level-dependent matrix
HR: Hazard ratio
HBV: Hepatitis B virus
HCV: Hepatitis C virus
HCC: Hepatocellular carcinoma
HKLC: Hong Kong Liver Cancer
INR: International normalized ratio
ICC: Intraclass correlation coefficient
LASSO: Least absolute shrinkage and selection operator
mRECIST: Modified Response Evaluation Criteria In Solid Tumors
NGTDM: Neighboring gray tone difference matrix
OS: Overall survival
PR: Partial response
PLT: Platelet
PD: Progressive disease
PFS: Progress-free survival
ROC: Receiver operating characteristic curve
ROIs: Regions of interest
SD: Stable disease
TBIL: Total bilirubin
TACE: Transarterial chemoembolization
