# Supplementary material for: CT texture analysis in predicting treatment response and survival in patients with hepatocellular carcinoma treated with transarterial chemoembolization using random forest models
Source: BMC Cancer. 2023 Mar 3;23:201. doi: 10.1186/s12885-023-10620-z (PMC9983241; doi:10.1186/s12885-023-10620-z)

**Suppl 1.** Contrast-enhanced CT in a 65-year-old male with HCC. The red mask is the demonstration of ROIs placement on the axial phase image.


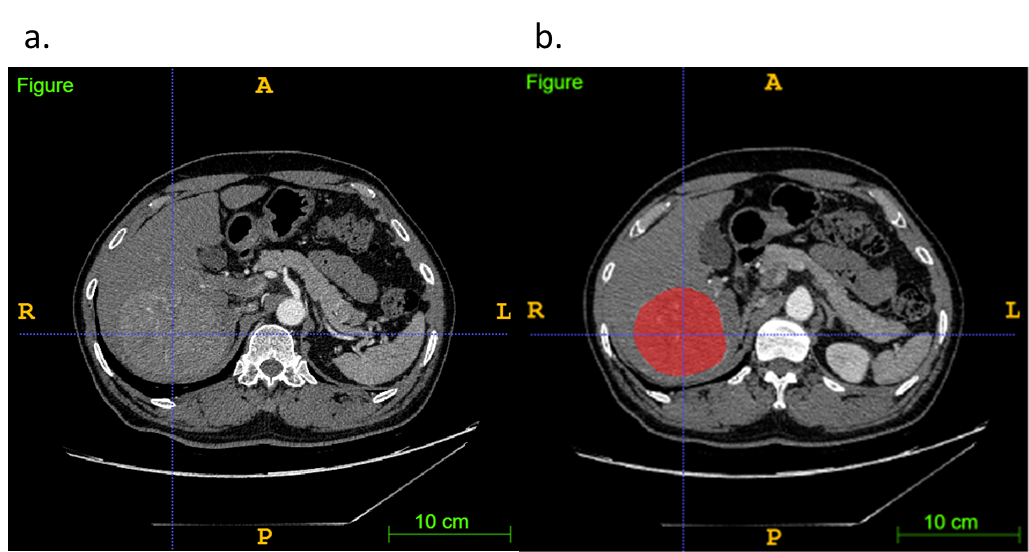


ROIs = regions of interest

**Suppl 2.** Summary of general imaging features

|  |  | Whole cohort | Progress-free cohort | Progress cohort | *p* |
| --- | --- | --- | --- | --- | --- |
| *N* |  | 289 | 224 | 65 |  |
| Largest tumor diameter* (cm) |  | 9.5  (1.5-21.1) | 8.5  (1.5-16.8) | 9.8  (1.8-21.1) | 0.167 |
| Number of lesions | Single lesion | 105 | 78 (74.3 %) | 27 (25.7 %) | 0.322 |
|  | Multiple lesions | 184 | 146 (79.3 %) | 38 (20.7 %) |  |
| The presence or absence of portal vein thrombus | Presence | 183 | 129 (70.5 %) | 54 (29.5 %) | **< 0.001** |
|  | Absence | 106 | 95 (89.6 %) | 11 (10.4 %) |  |
| The presence or absence of ascites | With ascites | 227 | 175 (77.1 %) | 52 (22.9 %) | 0.746 |
|  | Without ascites | 62 | 49 (79.0 %) | 13 (22.5 %) |  |

* Non-normal distribution summarised as median and range

**Suppl 3.** Feature selection using the LASSO algorithm. (A) Selection of the tuning parameter (λ) using 10-fold cross-validation and the minimum criteria. A plot of the partial likelihood deviance was made against log (λ). The minimum and 1-SE criteria were used to draw the dotted vertical lines at the optimal values. (B) Profiles of the LASSO coefficients for the texture features. The vertical line was drawn at a value selected from the log (λ) sequence using 10-fold cross-validation. Seventeen texture features were selected within this range.


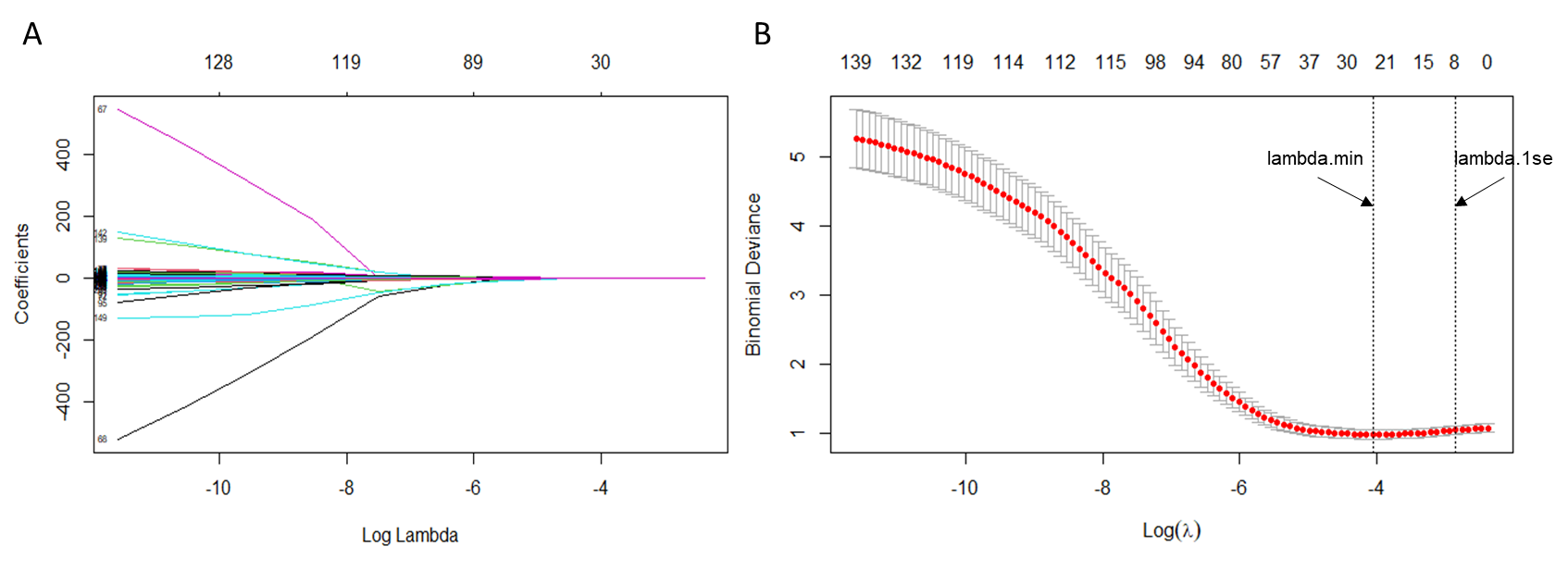


LASSO = least absolute shrinkage and selection operator

**Suppl 4.** ROC curves of Model 1 (A) incorporating selected clinical information, general imaging features and texture features and Model 2 (B) without texture features for the prediction of treatment response.


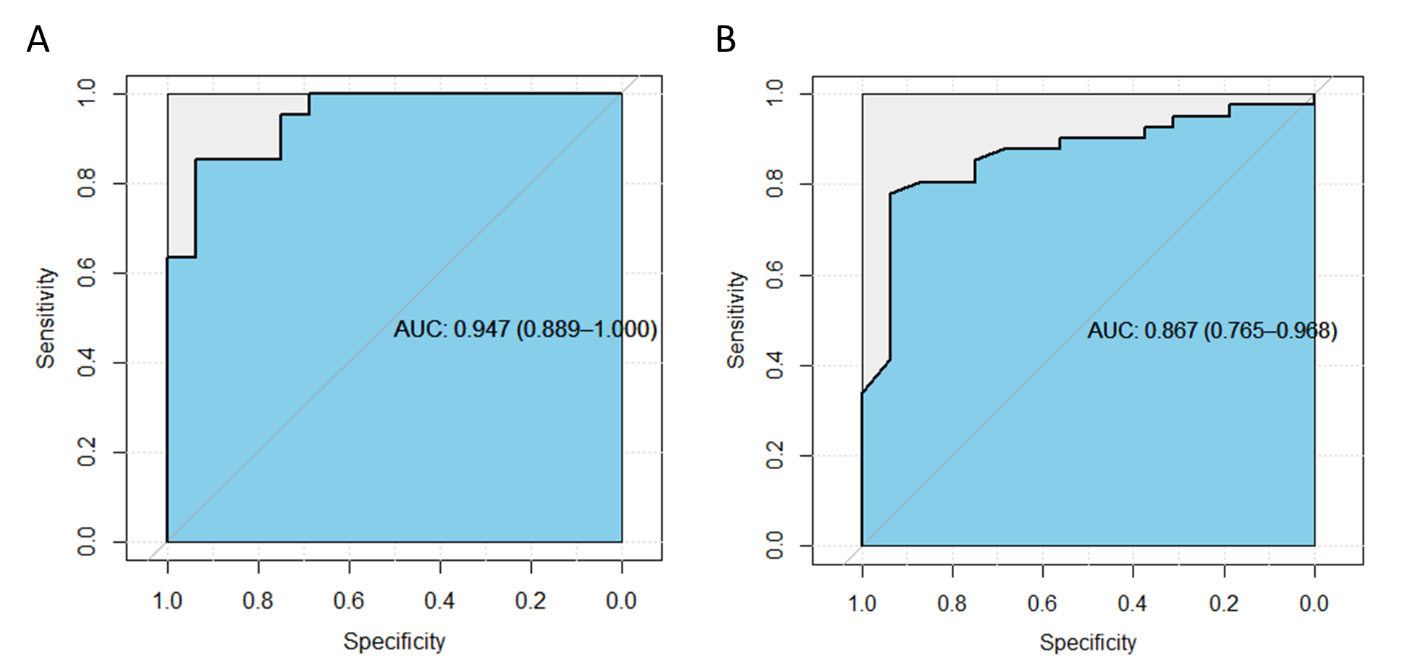

Supplement: Supplementary file 1 — Additional file 1: Suppl 1. Contrast-enhanced CT in a 65-year-old male with HCC. The red mask is the demonstration of ROIs placement on the axial phase image. Suppl 2. Summary of general imaging features. Suppl 3. Feature selection using the LASSO algorithm. (A) Selection of the tuning parameter (λ) using 10-fold cross-validation and the minimum criteria. A plot of the partial likelihood deviance was made against log (λ). The minimum and 1-SE criteria were used to draw the dotted vertical lines at the optimal values. (B) Profiles of the LASSO coefficients for the texture features. The vertical line was drawn at a value selected from the log (λ) sequence using 10-fold cross-validation. Seventeen texture features were selected within this range. Suppl 4. ROC curves of Model 1 (A) incorporating selected clinical information, general imaging features and texture features and Model 2 (B) without texture features for the prediction of treatment response. [file 12885_2023_10620_MOESM1_ESM.docx]
